# Clinical Outcome Features of Non-HIV Kaposi's Sarcoma and the Role of Wide Excision on Survival: A Retrospective Cohort Study

**DOI:** 10.1097/SAP.0000000000004189

**Published:** 2025-03-03

**Authors:** Chia-Kai Hsu, Fang-Yu Hsu, Hung-Chi Chen, Chang-Cheng Chang

**Affiliations:** From the a Division of Plastic Surgery, Department of Surgery, China Medical University Hospital, Taichung; b Division of Plastic Surgery, Department of Surgery, Chang Gung Memorial Hospital, Chiayi, Taiwan.

**Keywords:** Kaposi's sarcoma, non-HIV, surgery, nonsurgical procedures

## Abstract

This single-center retrospective study enrolled 22 patients, who were divided into two groups: those who underwent wide excision (group A) and those who did not (group B). Group A included 13 patients, and group B included 9. Most of the cases fell into early stages (I and II). In group A, six patients solely underwent wide excision, whereas seven received additional treatments such as radiotherapy or chemotherapy. Conversely, in group B, four patients received chemotherapy, four received radiotherapy, and one was managed through observation alone. The mean follow-up times were 60.15 and 43.44 months in groups A and B, respectively, with no significant difference observed. In group A, nine patients died, whereas, in group B, six died during the follow-up period. Lesion-free intervals after surgery were observed predominantly in group A, whereas, in group B, seven patients never experienced freedom from skin lesions. Kaplan-Meier survival curve analysis demonstrated a significant difference between the survival curves of the two groups (*P* = 0.046). Group A exhibited a more favorable outcome in the survival curve analysis. This significant difference could be attributed to the higher survival rates observed in group A during the first 5 years of follow-up. This finding underscores the potential benefit of surgical intervention in the management of non-HIV Kaposi's sarcoma, particularly in the short- to medium-term survival outcomes. Among patients with non-HIV Kaposi's sarcoma, especially those in the early stages, wide excision appears to play a role in treatment.

## Abstract 2

The standard treatment for non-HIV Kaposi's sarcoma is not yet well-established. Although various treatment modalities exist, their efficacy remains uncertain. However, certain patients undergoing surgical intervention have shown promising outcomes. This study aimed to assess the efficacy of wide excision in patients with non-HIV Kaposi's sarcoma and evaluate their clinical outcomes.

Skin lesions in Kaposi's sarcoma typically manifest as painless macules with a purple hue.^1,2^ Non-HIV Kaposi's sarcoma is primarily associated with HHV8 infection, particularly in individuals with immunocompromised status.^3^ Apart from acquired immunodeficiency syndrome (AIDS)–associated Kaposi's sarcoma, other types include class (sporadic), endemic (African), and iatrogenic (immunosuppression-related) types, which are relatively uncommon in clinical practice.^4^

Regarding treatment protocols outlined in the literature, therapies for HIV-positive cases adhere to standard rules and the National Comprehensive Cancer Network (NCCN) guidelines.^5^ Treatment approaches for Kaposi's sarcoma vary based on lesion classification. For asymptomatic limited cutaneous lesions, observation along with antiretroviral therapy (ART) is recommended initially, whereas symptomatic limited cutaneous lesions may require ART combined with topicals, systemic therapy, intralesional chemotherapy, radiotherapy, or local excision. Advanced cutaneous, oral, visceral, or nodal lesions may necessitate ART along with radiotherapy, systemic therapy, or participation in clinical trials.^6–8^ However, no standardized treatment protocol has been established for non-HIV Kaposi's sarcoma.^9^

In general, elective surgery becomes an option when localized skin lesions become problematic and surgical intervention is feasible.^10^ Therefore, this study aimed to assess the outcomes in patients with non-HIV Kaposi's sarcoma treated surgically to identify meaningful disparities in surgery-related treatment outcomes.

## MATERIALS AND METHODS

This single-center, pragmatic, retrospective study compared outcomes among patients with non-HIV Kaposi's sarcoma treated with wide excision or those who were not treated with surgery.

**FIGURE 1:**
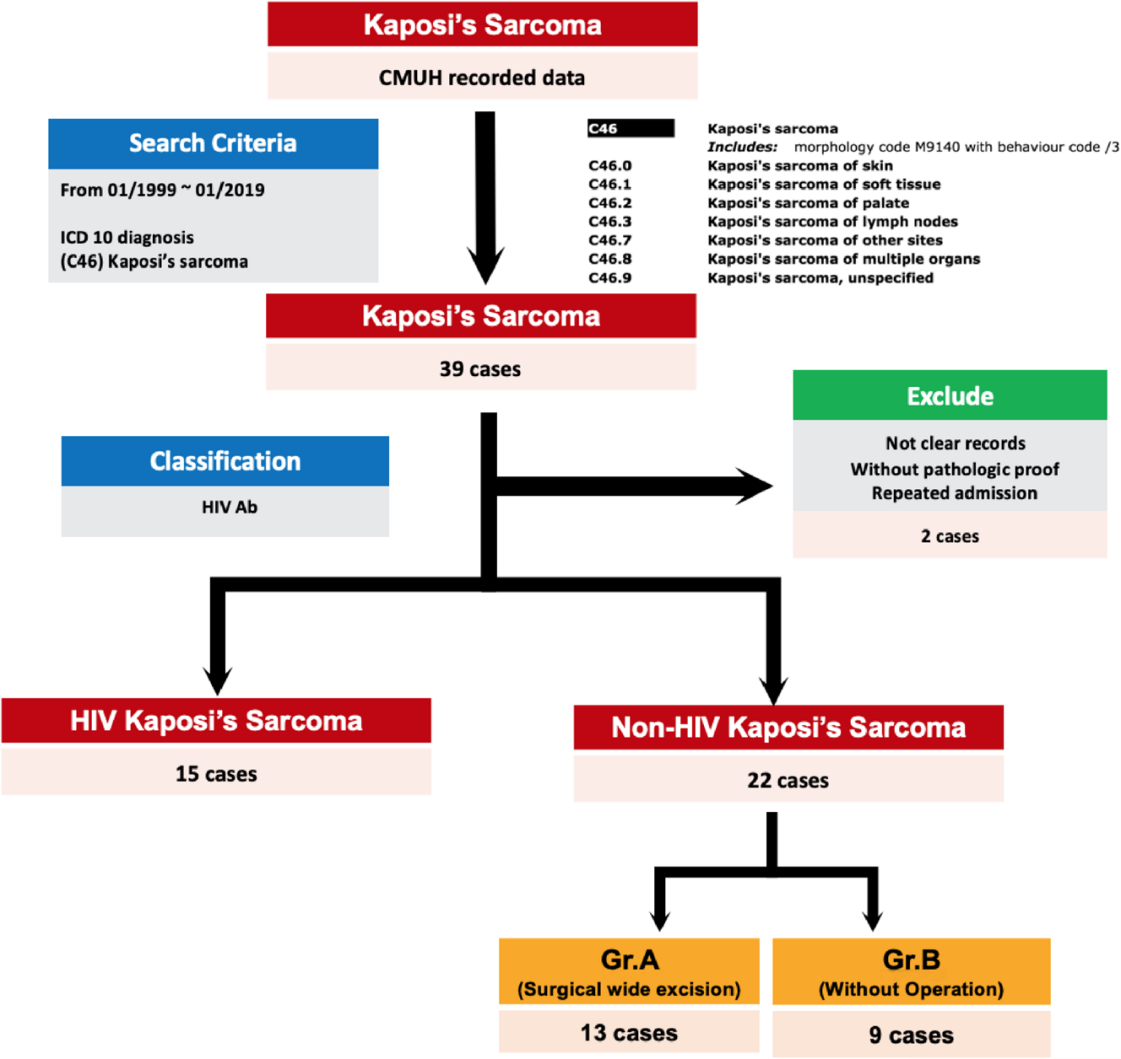
Enrolled patients with ICD diagnosis (C46) and pathological records between 1990 and 2023. The patients were divided according to the results of the HIV test and then to whether they underwent or did not undergo surgery.

Patients with an ICD diagnosis (C46: Kaposi's sarcoma) at CMUH between 1990 and 2023 were screened. Over 30 years, 39 patients were initially identified. After excluding 2 patients because of inadequate data to avoid misinterpretation, 37 patients remained. They were then divided into two groups based on their HIV status: Kaposi's sarcoma with HIV and without HIV. Among them, 22 patients had non-HIV Kaposi's sarcoma (Fig. 1).

Basic data from these patients, including age, sex, comorbidities, diagnosed Kaposi's sarcoma data, Kaposi's sarcoma lesion site and features, staging (European Journal of Dermatology staging), immunosuppressive status, associated disorders, performance status at the time of diagnosis, treatment modalities (chemotherapy, radiotherapy, operation, and combined therapy), follow-up time, symptom-free time, response to treatment, and cause of death if the patient died, were collected.

The 22 patients with non-HIV Kaposi's sarcoma were further categorized into two groups based on whether they underwent wide excision or not. Wide excision surgery with at least 2-cm margins was performed. Consequently, 13 patients had undergone surgery for Kaposi's sarcoma (group A), whereas the remaining 9 patients had not undergone surgery (group B) (Fig. 1). The differences in the outcomes between the two groups, particularly regarding the follow-up period and survival rate, were compared.

A two-sided *P* value of <0.05 was considered to indicate a statistically significant outcome. Analyses were performed using SPSS version 28, and survival curves were compared using the log-rank test.

## RESULTS

As previously mentioned, patients with non-HIV Kaposi's sarcoma accounted for 59% (22/37) of all Kaposi's sarcoma cases. Of these 22 patients with non-HIV Kaposi's sarcoma, 90.9% were male, with a mean age at diagnosis of 69.7 years. In addition, 90.9% (20/22) of the patients had lesions located at the lower extremities, and 68% (15/22) of them died during the follow-up period, which continued until loss to follow up or death.

All clinical characteristics and outcome analyses are presented in Table 1. In group A (n = 13), seven patients were in stage I, five were in stage II, and one was in stage IV. In group B (n = 9), two patients were in stage I, five were in stage II, one was in stage III, and one was in stage IV. The distribution of stages was comparable between the two groups, and the difference was not statistically significant, with most cases falling into early stages (I and II). Associated skin lesions and treatment methods are outlined in Table 2. In group A, 11 patients had a Karnofsky scale score of >70, one scored 50 to 70, and one scored <50. In group B, five patients had a Karnofsky scale score of >70, one scored 50 to 70, and three scored <50.

**TABLE 1:** Clinical Characteristics and Outcomes

Clinical Characteristics	Gr. A (N = 13)	Gr. B (N = 9)	*P* Value
Sex			0.075
Male	13 (100%)	7 (77.78%)	
Female	0 (0%)	2 (22.22%)	
Mean diagnosed age (year)	67.84 ± 11.00	72.44 ± 8.91	0.503
Staging			0.581
I	7	2	
II	5	5	
III	0	1	
IV	1	1	
Karnofsky performance			0.067
>70	11	5	
50–70	1	1	
<70	1	3	
Other treatment modalities			
Radiotherapy	5	4	
Chemotherapy	3	4	
Disease status after therapy			0.447
Complete response	8	2	
Partial response	3	4	
Poor response	2	3	
Follow-up period			
Mean follow-up time (month)	60.15 ± 42.26	43.44 ± 49.92	0.796
Median follow-up time (month)	61	33	
Death	9	6	0.582

**TABLE 2:** Treatment of Non-HIV Kaposi's Sarcoma With Surgical Intervention

Cases	KP Site	Lesions	Initial Skin Lesion Numbers	Surgery	Margin Free	Combined Treatment	Postop Complications	Symptoms Free Period (months)
1	Right leg	Disseminated	3	Wide excision + skin graft	+	Radiotherapy	−	47
2	Right sole	Localized	1	Wide excision + skin graft	+	Radiotherapy	−	23
3	Bilateral legs	Localized	3	Wide excision	N/A	Radiotherapy	Wound site verrucous plantaris	60
4	Left plantar foot	Localized	1	Wide excision + skin graft	+	−	−	19
5	Bilateral soles	Disseminated	2	Wide excision + skin graft	−	−	−	19
6	Left foot	Localized	1	Wide excision + free flap	−	−	Wound infection	60
7	Left sole	Disseminated	1	Wide excision + skin graft	−	Radiotherapy	−	15
8	Left sole	Localized	1	Wide excision	N/A	Chemotherapy	−	1
9	Right leg	Localized	1	Wide excision	+	−	−	90
10	Right leg	Localized	2	Wide excision + skin graft	+	Chemotherapy	−	5
11	Left foot	Localized	2	Wide excision	+	−	−	20
12	Right 2nd toe	Localized	1	Wide excision	N/A	Radiotherapy + chemotherapy	−	Never free
13	Left foot	Localized	1	Wide excision	+	−	−	82

Regarding therapeutic methods, in group A, six patients underwent wide excision alone, one also received radiotherapy and chemotherapy, two also received chemotherapy, and four also received radiotherapy. In group B, four patients received chemotherapy, four received radiotherapy, and one did not receive any treatment for Kaposi's sarcoma because of other underlying diseases with higher priority and was subsequently lost to follow-up.

Regarding follow-up periods, the median follow-up and mean follow-up times were 61 and 60.15 months in group A, and the median follow-up and mean follow-up times were 33 and 43.44 months in group B, respectively. No significant difference was found in the mean follow-up time (*P* = 0.796).

In terms of outcomes, a higher complete response rate was observed in group A (61.5% complete response, 23% partial response, and 15.3% poor response) than in group B (22% complete response, 44% partial response, and 33% poor response). Mortality rates were comparable between the two groups, with various causes including pancreatic cancer, septic shock, stroke, and unknown reasons.

Regarding postoperative complications, only one patient in group A experienced wound site cellulitis, and another patient suffered from wound site verrucous plantaris. As for symptom-free time, 10 of 13 patients in group A experienced symptom-free periods after undergoing wide excision, whereas only two had symptom-free periods after treatment in group B. Most patients in group B who did not undergo wide excision continued to experience symptoms.

**FIGURE 2:**
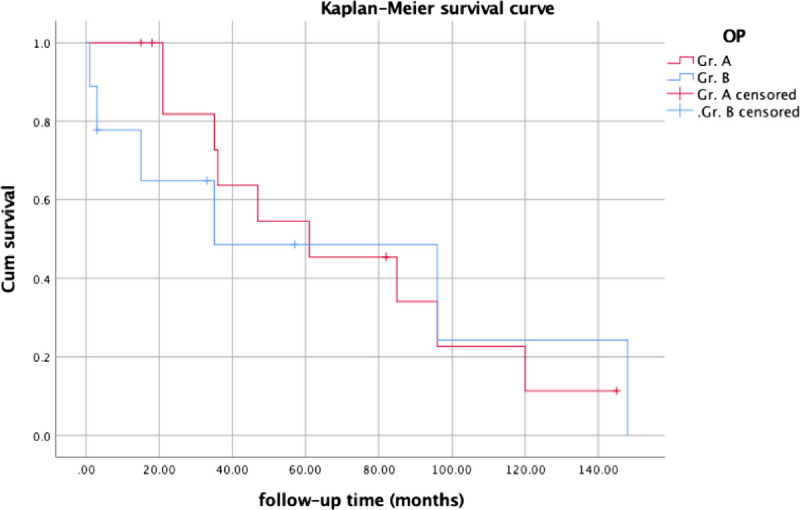
Kaplan-Meier curve of the two non-HIV Kaposi's sarcoma groups, group A with wide excision and group B without wide excision.

In evaluating prognosis among patients with non-HIV Kaposi's sarcoma, follow-up periods and survival rates were compared between the groups using Kaplan-Meier curves (Fig. 2). Statistical analysis revealed a significant difference (*P* = 0.046) in survival rates by the log-rank test possibly because of the higher survival rates noted during the first 5-year follow-up period in group A.

## DISCUSSION

Histologically, during skin lesion formation in Kaposi's sarcoma, thin-walled vascular spaces are visible in the upper dermis, accompanied by a sparse mononuclear cell infiltrate comprising lymphocytes, plasma cells, and macrophages. Subsequently, spindle cell bundles accumulate around areas of angioproliferation. Larger fascicles of spindle-shaped endothelial cells accumulate, resulting in fewer and more compact vascular slits, leading to the development of well-defined nodules and a more solid tumor. This histological progression may explain why skin lesions in patients with non-HIV Kaposi's sarcoma are more limited and localized.^11^ In addition, lymphangioma-like skin lesions are more common in non-HIV Kaposi's sarcoma, consisting of networks of irregularly dilated lymphatics lined by flat and cytologically banal endothelial cells, presenting as compressible nodules resembling fluid-filled cysts.^12^

Because of the scarcity of non-HIV Kaposi's sarcoma, published treatment options are primarily based on retrospective studies and case reports. Consequently, no treatment guidelines have been established for this condition. The relative rarity of the disease, presence of comorbidities limiting treatment options, and challenges in participation in clinical trials make it challenging to conduct prospective randomized trials comparing different treatments. Therefore, patients with non-HIV Kaposi's sarcoma typically undergo treatments based on the attending physician's experience and individual response.^13,14^ Common therapies include chemotherapy, radiotherapy, and combined operation for localized skin lesions.

In some literature reviews, the overall 1-, 5-, and 10-year survival rates for non-HIV Kaposi's sarcoma were reported as 92%, 69%, and 46%, respectively, after diagnosis, with a median survival time of 9.6 years.^15^ The persistent disease rate was 15%, and the disease-free rate was 35% in median 6-year follow-up.^16,17^ However, a significant gap exists in treatment outcomes among patients with non-HIV Kaposi's sarcoma. The leading causes of death in these patients include secondary malignancy (24%) and iatrogenic diseases (22%).^11,12^ However, because of the small number of non-HIV Kaposi's sarcoma cases, the results between various studies may not be consistent.

Most literature suggests that non-HIV Kaposi's sarcoma stages I to III are categorized behaviorally as nonaggressive or locally aggressive, with only stage IV considered aggressive.^18^ In our study, both group A and group B each have only one case of stage IV, with the majority being cases of stages I and II. It seems that, among patients with non-HIV Kaposi's sarcoma, especially those in the early stages, wide excision may be beneficial in treatment.

Regarding performance status assessment, the Karnofsky scale was chosen because of its precision in reflecting physical status compared with the ECOG scale. The cases were categorized into three groups based on their Karnofsky scores: >70, 50 to 70, and <50. A Karnofsky score of >70 indicates normal activities with effort and is commonly used to evaluate the stage of HIV-related Kaposi's sarcoma. This score has been identified as an important prognostic factor in HIV-related Kaposi's sarcoma in some literature. On the contrary, a Karnofsky score of <50 signifies that patients require considerable assistance and frequent medical care, marking a critical turning point in the patient's condition.

About the Kaplan-Meier curve, the statistical analysis revealed a *P* value of 0.046, indicating a significant difference in the survival curve. This significant difference could be attributed to the higher survival rates observed in group A during the first 5 years of follow-up. This suggests that patients who underwent surgery for non-HIV Kaposi's sarcoma may experience better outcomes and higher survival rates in the initial 5 years after treatment. This finding underscores the potential benefit of surgical intervention in the management of non-HIV Kaposi's sarcoma, particularly in the short to medium-term survival outcomes.

In addition, most patients in group A experienced monthly symptom-free time. However, only a few studies have discussed the relationship between non-HIV Kaposi's sarcoma and surgery. Despite the lack of comprehensive research in this area, the findings of this study suggest that elective surgery may lead to better outcomes and higher survival rates in patients with non-HIV Kaposi's sarcoma.^17^ Therefore, surgical intervention for non-HIV Kaposi's sarcoma should be considered a crucial treatment.

Although the NCCN guidelines exclusively offer treatment recommendations for AIDS-related Kaposi's sarcoma, certain therapies outlined in these guidelines may serve as a reference for non-HIV Kaposi's sarcoma. Elective surgery combined with chemotherapy or radiotherapy could be an effective treatment approach for non-HIV Kaposi's sarcoma. Although these recommendations are not explicitly specified for non-HIV Kaposi's sarcoma, the principles of treatment for Kaposi's sarcoma may be applicable across various contexts, including non–HIV-related cases. However, individualized treatment plans should be formulated based on the specific characteristics of each patient and disease extent. Further research and clinical studies are warranted to determine the efficacy and optimal treatment strategies for non-HIV Kaposi's sarcoma.

This study has several limitations that warrant consideration. First, it was conducted at a single center, which may limit the generalizability of the findings to broader populations. Second, the study included a relatively small number of cases, which could affect the statistical power and reliability of the results. Third, the pragmatic study design relied on data collection from electronic medical records, which may have led to limitations in obtaining detailed patient characteristics and clinical information. This could potentially introduce outcome ascertainment bias and affect the accuracy of the results. Fourth, the small number of cases may have influenced the statistical analysis, making it challenging to detect significant differences between groups. Fifth, the patients were managed by different attending physicians, potentially resulting in variations in the management and assessment of Kaposi's sarcoma lesions. Sixth, the duration of follow-up for survival rate assessment may be a limitation. The cutoff observation days were determined based on the last outpatient clinic visit, which could result in varying lengths of follow-up among the cases. This variability in follow-up duration could affect the statistical power of the analysis and the interpretation of the results.

In summary, although this study provides valuable insights into the treatment of non-HIV Kaposi's sarcoma, its limitations highlight the need for larger multicenter studies with longer follow-up periods to further elucidate the efficacy and outcomes of different treatment approaches. Further investigation and larger studies are needed to validate and better understand the potential benefits of surgery in the management of non-HIV Kaposi's sarcoma.

## CONCLUSIONS

This retrospective study of non–HIV-infected adults who underwent wide excision surgery or received alternative modalities did not demonstrate a significant difference in the primary outcome between patients who underwent surgery and those who did not. However, its notable findings included a relatively lower death rate in the early periods and better outcomes in terms of being symptom-free time in group A patients who underwent wide excision. These findings may be attributed to the potentially better clinical status of patients in group A, characterized by more localized skin lesions and a more suitable physical condition for surgical intervention. Most cases are in the early stages, which may also have an impact. In addition, patients who underwent wide excision instead of radiotherapy or chemotherapy may have experienced fewer side effects and discomfort associated with these alternative therapies.

Although many retrospective studies have reported long-term experiences following patients with standardized conditions, these findings may not directly apply to patients with non-HIV Kaposi's sarcoma. However, the need for guidelines and standardized techniques in the management of non-HIV Kaposi's sarcoma is increasingly recognized. We hope that the wider adoption of such guidelines will lead to greater standardization of the staging criteria and management protocols for the treatment of non-HIV Kaposi's sarcoma in the future.
